# Parsing Model and a Rational Theory of Memory

**DOI:** 10.3389/fpsyg.2021.657705

**Published:** 2021-06-23

**Authors:** Jakub Dotlačil, Puck de Haan

**Affiliations:** ^1^Utrecht Institute of Linguistics, Utrecht University, Utrecht, Netherlands; ^2^Artificial Intelligence, University of Amsterdam, Amsterdam, Netherlands

**Keywords:** computational psycholinguistics, cognitively constrained parsers, memory retrieval, rational theory of memory, modeling reading data

## Abstract

This paper explores how the rational theory of memory summarized in Anderson (1991) can inform the computational psycholinguistic models of human parsing. It is shown that transition-based parsing is particularly suitable to be combined with Anderson's theory of memory systems. The combination of the rational theory of memory with the transition-based parsers results in a model of sentence processing that is data-driven and can be embedded in the cognitive architecture Adaptive Control of Thought-Rational (ACT-R). The predictions of the parser are tested against qualitative data (garden-path sentences) and a self-paced reading corpus (the Natural Stories corpus).

## 1. Introduction

In the rational theory of cognition, it is argued that cognitive functions are largely shaped by our adaptation to the environment. In this view, it is assumed that various aspects of our behavior can be explained as the result of the optimization to the structure of the environment. The rational theory of cognition has been fruitful in explaining regularities in categorization, learning, communication and reasoning, among others (Anderson, 1990, 1991; Oaksford and Chater, 1994, 2007; Tenenbaum et al., 2011; Franke and Jäger, 2016; Piantadosi et al., 2016).

One particularly successful case of the rational theory was its application to the study of human memory, as summarized in Anderson (1991). Assuming that the human memory should strive to provide information that is needed at a particular situation and that it is costly and takes time to retrieve elements from memory, we would expect that the retrieval of an element be related to the probability that it is needed. That is, elements that are most likely to be needed at a particular situation will be prioritized in retrieval. Since retrieval is ordered by need probabilities, it is expected that less needed items require more time to be recalled. Furthermore, if retrieval is abandoned when the cost for retrieval exceeds some threshold, we expect the less needed an item is, the more likely it is that its recall fails. These predictions have been largely confirmed, see Anderson (1991).

The rational theory of memory played an important role in the development of the cognitive architecture Adaptive Control of Thought-Rational, ACT-R (Anderson and Lebiere, 1998; Anderson et al., 2004), which in turn played an important role in psycholinguistic models of parsing (Lewis and Vasishth, 2005; Lewis et al., 2006; Reitter et al., 2011; Engelmann et al., 2013; Vogelzang et al., 2017; Brasoveanu and Dotlačil, 2020). Lewis and Vasishth (2005) and subsequent works showed, in particular, that the rational theory of memory implemented in ACT-R is insightful in analyzing the pattern of recall in forming dependencies during parsing, for example, subject-verb dependency as in (1-a) and antecedent-reflexive dependency as in (1-b) (see also Lewis et al., 2006; Van Dyke, 2007; Wagers et al., 2009; Dillon et al., 2013; Kush et al., 2015; Lago et al., 2015; Jäger et al., 2017; Jäger et al., 2020; Nicenboim et al., 2018; Villata et al., 2018; Engelmann et al., 2019; Vasishth et al., 2019; Smith and Vasishth, 2020, among others).


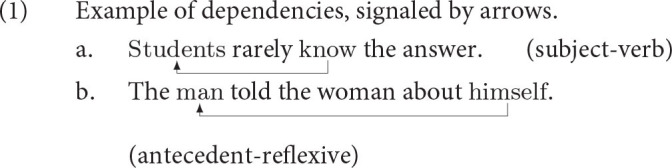


This brings us to the research topic of this paper, namely, studying whether other aspects in which parsing has to rely on memory can also be seen as fitting the research programme of the rational theory of cognition. In particular, during comprehension and production, native speakers have to continuously rely on their past knowledge of parsing rules. For instance, in (1), readers would not be able to comprehend the sentences correctly unless they recall that subjects normally precede verbs in English, verbs are followed by objects, English has prepositions (not post-positions) etc. From the perspective of the rational theory of memory, it is expected that the retrieval of parsing rules, such as these should follow the general considerations highlighted above, i.e., parsing rules should be retrieved in the order of their need probability and the order should monotonically correlate with latencies and accuracies. We will show that it is indeed possible to construct parsing on the basis of the rational theory of memory. The resulting model can furthermore correctly predict qualitative data in psycholinguistics (garden-path phenomena) and its predictions match behavioral measures in a psycholinguistic corpus (Natural Stories Corpus, Futrell et al., 2018).

The structure of the paper is as follows: in the following section, we briefly introduce the rational theory of memory as part of the cognitive architecture ACT-R. Next, we present transition-based parsers developed in computational linguistics and show how transition-based parsing and cognitive architectures can be combined. The cognitively informed parser is then evaluated on garden-path examples and data from Natural Stories Corpus. Finally, our research is briefly compared to related works in computational psycholinguistics.

## 2. Modeling Memory Retrieval in Rational Theory

Adaptive Control of Though-Rational assumes, true to its name, that various cognitive functions should be modeled as a case of rational theory of cognition. Here, we will focus on how memory and memory retrieval are formalized in ACT-R.

ACT-R assumes two types of memory: procedural memory and declarative memory. We focus here on the latter, the declarative memory, which is used for the storage of factual knowledge.^1^

The goal of the declarative memory system should be to recall a piece of information *i* that is needed to achieve the current goal. As is common in ACT-R, we will formalize pieces of information as chunks. These are attribute-value matrices, or, in the terminology of ACT-R, slot-value matrices. An example of a chunk, representing a simplified piece of information retrieved in the dependency in (1-a), is shown in (2). In this notation, slot names appear on the left side and their values on the right side. The chunk represents the knowledge that a plural subject of the form *students* was encountered and stored in memory.


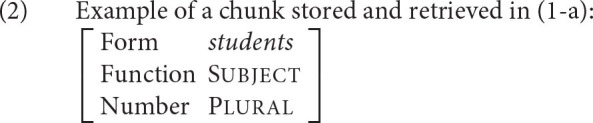


Assuming that retrieving a chunk is costly and takes time, retrieval from memory must be constrained. An optimal retrieval system would prioritize those chunks that are more likely needed for the current goal. In general, it should hold that the recall of a piece of information, chunk *i*, adjusted by the value of the current goal *G* should not exceed the cost of the retrieval *C*.


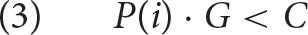


The task of the rational theory of memory is to find a reasonable estimation of *P*(*i*). In ACT-R, it is assumed that *P*(*i*), the probability that *i* is needed, is conditionalized on two sources of information: (i) the history *H*_*i*_, that is, the past use of *i*;and (ii) the current context *Q*. We thus need to estimate *P*(*i*|*H*_*i*_, *Q*), which can be easily done using Bayes' rule. However, rather than expressing the conditional probability directly, it is standard in ACT-R to estimate log-odds. The estimation is expressed in (4) (*i*^*c*^ is the complement of *i*, i.e., *P*(*i*^*c*^) is the probability that *i* is not needed; *Q*, the current context, consists of indices *j*, which we call cues).


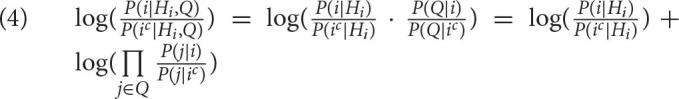


The inference in (4) makes the common assumption that while the probability that *i* is being needed is dependent on *H*_*i*_ and *Q*, the probabilities of the cues *j* in the current context *Q* are mutually independent and not dependent on the history *H*_*i*_, conditional on *i* (see Anderson, 1991). The log-odds in (4) have a special status in ACT-R. They are called the activation of *i*, written as *A*_*i*_. The activation consists of two parts: the history component [the first addend in(4) ] and the context component [the second addend in(4) ]. In ACT-R, the history component is called base-level activation, abbreviated as *B*_*i*_, and the context component is called spreading activation, which we will abbreviate as *S*_*i*_. We can rewrite the formula as follows^2^:


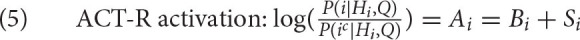


Let us see how ACT-R estimates the history and the context components. Before doing so, we want to stress two things. First, the theory we are to discuss is generally and widely accepted by the ACT-R research community. Second, it is important to realize that the estimations of both the history component and the context component are not just arbitrary equations that happen to fit memory data. They should reflect the estimations that the mind draws from the structure of the environment in order to arrive at the best estimation of *P*(*i*) used in (3), just as a rational theory of cognition would make us expect. However, we will not present evidence that the following estimations are generalized from the structure of the environment since this has been done elsewhere (see Anderson, 1991).

The base-level activation *B*_*i*_ of a chunk is given in (6) and captures the fact that the probability that a chunk will be used next time decreases as a power function of the time since the last use, but it is also affected by the number of times that the chunk has been used. The base-level activation is expressed as the log of the sum of $t_{k}^{-d}$, where *t*_*k*_ is the time elapsed between the time of presentation *k* and the time of retrieval. *d* is a negative exponent (decay), a free parameter of ACT-R, often set at its default value of 0.5. “Presentation” in ACT-R means two things. Either it refers to the moment that the chunk was created for the first time (i.e., someone learns a particular fact), or the moment when the chunk was successfully recalled from declarative memory to be used in some context, after which it is stored in declarative memory again.


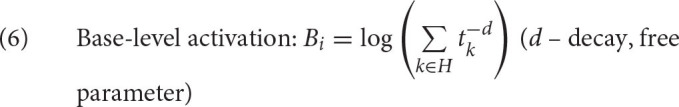


The second element in the calculation of activation is given in (7). To keep the calculation manageable, some simplifying assumptions are introduced (see Anderson, 1991; Anderson and Lebiere, 1998). First, it is assumed that the cues *j* in the current context are independent of each other (and of the history *H*_*i*_), conditional on *i*. Second, the denominator, which should be *P*(*j*|*i*^*c*^), is simplified into *P*(*j*) since conditionalizing *j* on the irrelevant piece of information *i*^*c*^ should not affect probabilities significantly and can be ignored. The resulting log of probability ratios, $\log\frac{P\left(j|i\right)}{P\left(j\right)}$ is called the associative strength and is standardly abbreviated as *S*_*ji*_. The equation also includes the weight *W*, which is a free parameter weighing the context component of the activation.


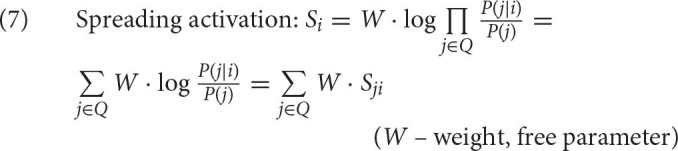


Finally, the equation in (8) shows how ACT-R estimates the associative strength *S*_*ji*_. This equation is only used if the cue *j* is predictive of the chunk *i*. If it is not, *S*_*ji*_ is set at 0. Simplifying somewhat, ACT-R assumes that a cue is predictive of a chunk if the cue appears as a value in the chunk.


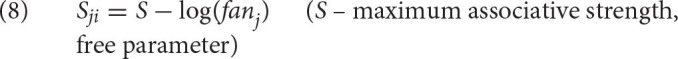


*S* is the log of the size of the declarative memory, but commonly, it is hand-selected as a large enough value to ensure that *S*_*ji*_ is always positive (see Bothell, 2017). fan_*j*_ is the number of chunks in memory that have the cue *j* as its value. For discussion as to why (8) approximates $\log\frac{P\left(j|i\right)}{P\left(j\right)}$, see Brasoveanu and Dotlačil (2020). It might also help to notice that the formula *S*_*ji*_ also expresses the following intuition: the associative strength (and consequently, activation) will be large when *j* appears only in a few chunks since in that case *j* is highly predictive for each of those chunks; the associative strength will decrease if there are more chunks in declarative memory that carry *j* as its value (see Anderson, 1974; Anderson and Lebiere, 1998; Anderson and Reder, 1999 for empirical evidence).

Finally, the formula in (9) shows how *A*_*i*_ is related to the time it takes to retrieve a chunk from declarative memory, *T*_*i*_. The relation between *A*_*i*_ and *T*_*i*_ is modulated by two free parameters, *F*, latency factor, and *f*, latency exponent.


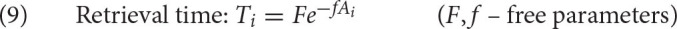


When both parameters are set at 1 (their default value), the retrieval time of a chunk *i* is simply the exponential of its negative activation, which is the reverse odds that the chunk *i* is needed in the current context [see (4)]:


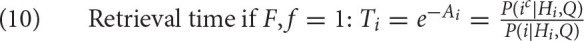


It follows from (10) that the more a chunk is needed to achieve the current goal, the faster it will be retrieved.

Let us illustrate how all the equations are put together on an example from the introduction, the subject-verb dependency.

Assume we comprehend or produce the verb in (11-a) and want the retrieve the chunk *students* to resolve the subject-verb dependency. For the purposes of this illustration, we assume that the chunk is represented in memory as shown in (11-b), repeated from (2). The dependency needs to be resolved for interpretational purposes since listeners need to know who the agent of *know* is. It is also necessary for production purposes since speakers need to know what inflectional form the verb should have.


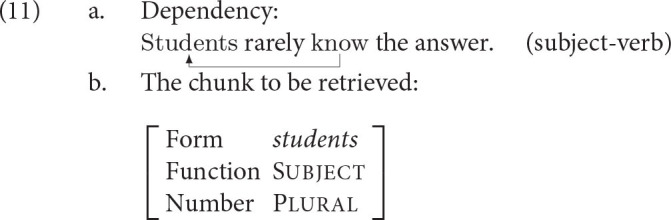


The activation of the subject *students*, its log-odds that the chunk is needed, consists of the base-level activation and the spreading activation. Suppose that 1 s elapsed since storing the chunk in memory and the chunk was not re-used. Then the base-level activation, calculated using the equation in (6), is:


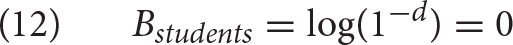


The spreading activation, calculated using the Equations (7) and (8), is given in (13). Note that the cues [subject], [plural] are the cues in the current context, i.e., we assume for this example that these two cues are present in the cognitive context when resolving the subject-verb dependency.


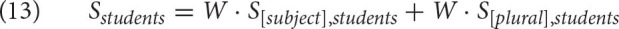


Let us assume that the free parameter *S* is set at 1 and so is the weight *W*. Since both cues appear in the chunk *students*, we have to calculate both addends as:


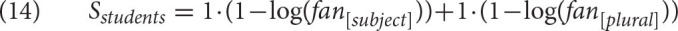


The only part that needs to be decided is the value of the fan for two cues. Let us assume that in the memory, there is no other subject and one other plural element. Then the calculation proceeds as follows:


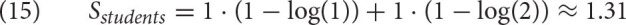


Finally, we can calculate retrieval times as follows:


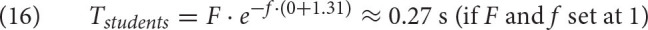


Based on the discussion of this example, one might note that the ACT-R model of declarative memory makes several predictions regarding retrieval times. Some of those are summarized in the bullet points below:

- The longer the time elapsed since a chunk was used last time, the lower base-level activation the chunk has. Consequently, chunks that were used a long time ago will be retrieved slower than chunks used recently.
- The less often a chunk was used, the lower base-level activation the chunk has. Consequently, chunks that are rarely used will be retrieved slower than chunks used often.
- The more a chunk matches cues of the current context, the higher the boost from spreading activation. Consequently, chunks with higher matches with cues should be retrieved faster.
- Increasing the fan of a cue will increase the time to retrieve an element. For example, imagine that more chunks with the value *plural* were stored in declarative memory. Then, the associative strength of any chunk with *plural* would be lower and consequently, it would take more time to retrieve such chunks.

To the extent that these qualitative predictions are confirmed, we have supporting evidence for the rational theory of memory as implemented in ACT-R. To the extent that quantitative predictions of the model can be well fit to retrieval data, we also have evidence that the estimates of the history and the context component of (4) in ACT-R are on the right track.

Various evidence has been collected showing that qualitative as well as quantitative predictions of the retrieval model in ACT-R are justified. Anderson (1991) and Anderson and Lebiere (1998) present supporting evidence from general cognitive tasks (independent of language). In psycholinguistics, Lewis et al. (2006), Jäger et al. (2017); Jäger et al. (2020), among others, summarize evidence that at least some cases of the retrieval of dependencies can be modeled as a case of ACT-R retrieval.

The goal of this paper is to apply the retrieval and memory model of ACT-R to a new domain. We will investigate how the rational theory of memory can model parsing knowledge and how the model of parsing can be embedded in ACT-R. We will show that once one thinks of parsing steps as chunks in declarative memory whose retrieval is driven by the same rules as other memory elements, the ACT-R model of memory becomes directly applicable to syntactic parsing. The activation that is associated with retrieved parsing steps can then be used to model the effect of context on processing, e.g., investigations that are mainly the domain of psycholinguistic parsing theories, such as the Surprisal Theory (Hale, 2001). To the extent that the resulting model of parsing makes correct quantitative and qualitative predictions, we construct evidence that processing difficulties observed during parsing can be approached from the vantage point of the rational theory of memory. The hypothesis explored in this paper is further investigated in Dotlačil (accepted)^3^, which also studies how individual components of ACT-R retrieval system affect the retrieval of parsing steps and how the retrieval of parsing knowledge interacts with the retrieval of dependencies in processing.

In section 3, we introduce transition-based parsing and show how such parsers can be built as a case of declarative memory in ACT-R. In section 4, we show how the model can be linked to reaction time data and evaluate its qualitative and quantitative predictions.

## 3. Transition-Based Parsing

We introduce transition-based parsers and show that they can be, to a large extent, embedded in ACT-R and combined with the memory structures discussed in section 2. Such a combination directly delivers behavioral predictions to be tested in the following sections.

Transition-based parsers are parsing systems that predict transitions from one state to another, following decisions made by a classifier. Since the classifier plays a crucial role in this type of parsers, these parsers are also called classifier-based parsers.

Transition-based parsers are most commonly implemented for dependency grammars and arguably, they are most successful and widespread when constructing dependency graphs (Nivre et al., 2007). However, they have also been applied to phrase-structure parsing (Kalt, 2004; Sagae and Lavie, 2005; Liu and Zhang, 2017; Kitaev and Klein, 2018, a.o.). This paper also develops a phrase-structure transition-based parser. We introduce a shift reduce variant of the transition-based parsing algorithm, which is arguably the most common type of transition-based parser for phrase structures, and show how it can be understood in terms of memory systems discussed in the previous section.

### 3.1. Algorithm of Transition-Based Phrase-Structure Parsing

The parsing algorithm works with two databases, a stack of constructed trees S and a stack of upcoming words with their POS (part-of-speech tags) W. When parsing begins, S is empty and W carries the upcoming words as they appear in the sentence, so that the first word appears at the beginning of the stack, followed by the second word, etc.

Parsing proceeds by selecting actions based on the content of S and W. Every parsing step P is a function from S,W to actions A, that is, P:S×W↝A. In the variant of the parser that we consider, there are three actions that the parser can select:

- shift
- reduce
- postulate gap

The first action, *shift*, pops the top element from the stack W and pushes it as a trivial tree onto stack S. An element in W is a pair 〈word, POS〉, the tree moved onto the stack is just the POS tag with the terminal the actual word.

The second action, *reduce*, pops the top element (if the reduction is unary) or it pops the top two elements (if the reduction is binary) in the stack of constructed trees S and creates a new tree. If the reduction is unary, the new tree has just one daughter under the root, the tree that was just popped from the stack. If the reduction is binary, the newly created tree has two daughters, the two trees that were just popped from the stack. In either case, the newly constructed tree is pushed on top of the stack S. It is assumed that all trees are at most binary, so no further reductions beyond binary reductions are necessary.

Finally, the third action, *postulate gap*, postulates a gap and resolves it to its antecedent. Not every parser in computational linguistics assumes this action, i.e., implemented parsers can proceed just by shifting and reducing (but see Crabbé, 2015; Coavoux and Crabbé, 2017a,b as examples of transition-based parsers that do consider gap resolution). We add gap resolution to our parser since ignoring gaps would make the parser less useful for psycholinguistics, which often studies the effect of gap resolution on processing.

There are several restrictions on the three actions. First, no shift can be applied when W is empty. When S is empty, no reduce can be applied and when it has only one tree, reduce binary cannot be applied. Finally, no more than two postulate gaps actions can be applied between two shifts. This last restriction ensures that the system does not fall into the infinite regress of gap postulation.

**Figure 1:**
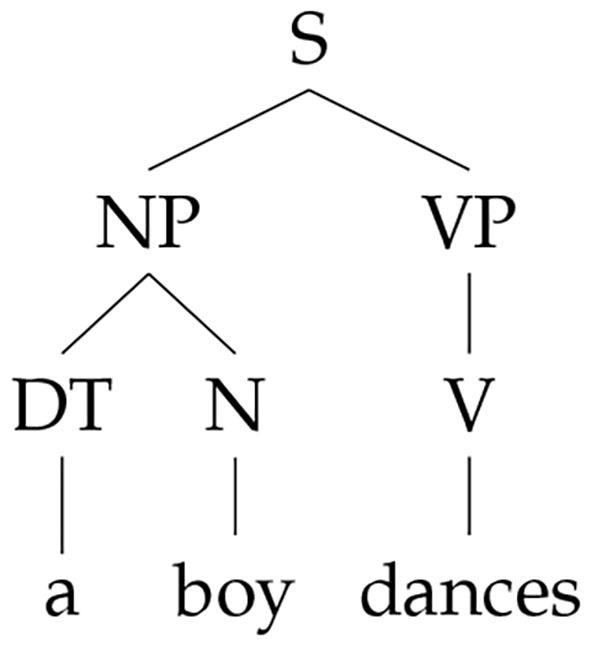
Phrase structure of *a boy dances*.

We illustrate the steps of the shift-reduce parser on a simple example: parsing of *a boy dances*. The phrase structure is shown in Figure 1 and the parsing steps are:


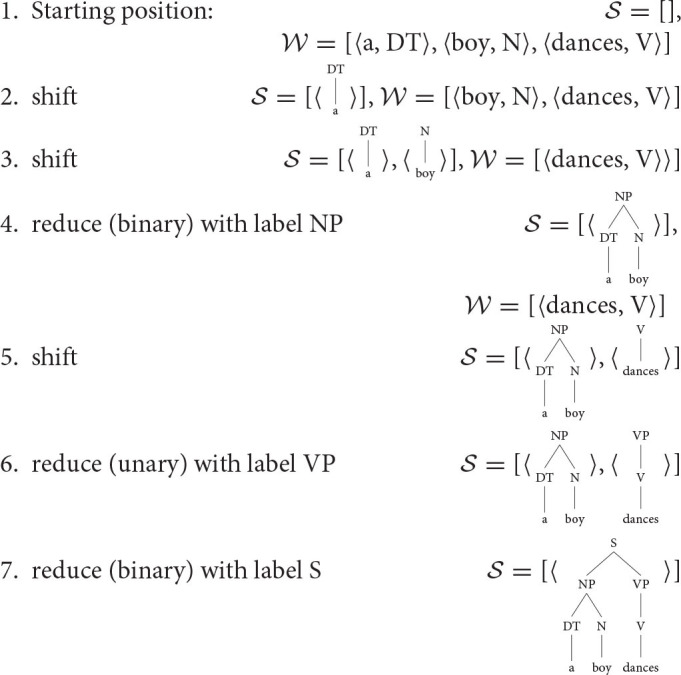


In this illustrative example, we assume that the parser knows what the right phrase structure is and parses toward that structure. Of course, the crucial question is what happens when the phrase structure is unknown and the parser needs to predict what action to take. This is discussed in the next section.

### 3.2. Parsing Steps as Memory Retrievals

The parsing step has to decide which action (among *shift, reduce*, and *postulate gap*) should be taken, and, if *reduce* is selected, how should the reduction be done: should it be unary or binary and what should the root label of the newly constructed tree be?

We investigate the hypothesis that the parsing step can be treated as a case of memory retrieval. The past parsing steps form the declarative memory of the parser. The parser retrieves a parsing step (or parsing steps) from memory that has the highest probability of being needed given the current goal. The current goal, in turn, is to parse the sentence. From this perspective, parsing is just a particular instantiation of rational theory of memory and can be embedded in ACT-R. The activation of a parsing step, i.e., the log-odds that a step is needed, is calculated from the history component and the context component. The former is derived from the time elapsed since the step has been used and re-used, the latter is calculated based on the cues in the current context and the spreading activation from these cues to chunks in declarative memory.

While it might be possible to think of the context as complete trees in S and all information in W, we will limit the amount of information in the two databases significantly. It will be assumed that S and W carry only some features about the trees and upcoming words, listed in (17). Thus, the parser itself never has a full snapshot of the phrase structure that it is deriving. It only carries some minimal, local information. The phrase structure can always be reconstructed through parsing steps the ACT-R agent (and, arguably, humans) took but there is no single snapshot in which all the information is available to the agent. This position is common in ACT-R parsing, see for example, Lewis and Vasishth (2005).


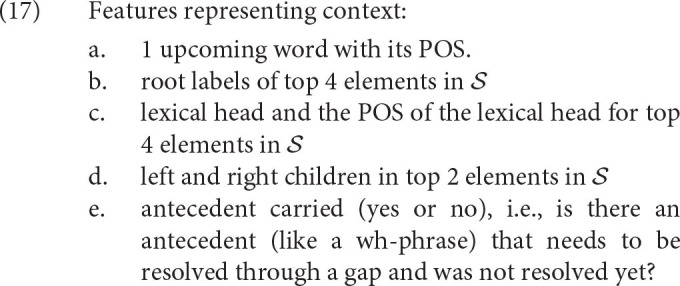


The features should be familiar, maybe with the exception of the lexical head. The head is a terminal that projects its phrase (a verb is the head of a verb phrase, a noun is the head of a noun phrase etc.; see Collins, 1997 on head projection in computational parsers, which this work follows).

All the features in (17) spread activation to chunks stored in declarative memory, which in turn represent all parsing steps completed in the past. Recalling the right parsing step is a case of memory retrieval that follows the rules in section 2. Consequently, it is predicted that different parsing steps might require different amounts of time depending on the time it takes to retrieve them. Parsing steps with higher activations will be recalled faster than parsing steps with lower activations. Activations, in turn, are based on the base-level activation and spreading activation, i.e., the ACT-R estimates of the history and the context component in calculating the need log-odds of a chunk.

## 4. Modeling Reading Data

We present an implementation of the model of sentence parsing built on the rational approach to memory and discuss two case studies testing the implementation.^4^ Section 4.1 introduces the model. Section 4.2 investigates whether the parser can predict processing difficulties for selected garden-path phenomena. Section 4.3 investigates whether the parser can be used to model self-paced reading time data from the Natural Stories Corpus (Futrell et al., 2018).

### 4.1. Parsing Model

We assume that a declarative memory consists of chunks that represent correct past parsing steps. These chunks are collected from the data in the Penn Treebank (PTB) (Marcus et al., 1993). As is standard, we split the section of the PTB data as follows: all the sections up to and including section 21 are used to train the parser, i.e., to collect the correct parsing steps; section 22 is used for development; section 23 is used to test the accuracy of the parser. Before training we pre-process and prepare the phrase structure by (i) transforming phrases into binary structures in the way described in Roark (2001) (see Roark, 2001; Sagae and Lavie, 2005 on the reasons to do), (ii) annotating phrases with head information, (iii) removing irrelevant information (coreference indices on phrases), (iv) lemmatizing tokens so that lexical heads are stored as lemmas, not as inflected tokens.

Parsing novel sentences consist of recalling the needed chunks, i.e., parsing steps collected from the PTB, from declarative memory. The recall is driven by the activation of the chunks. To calculate the activation of each chunk, formulas in section 2 are applied. We assume that the parser will recall the three chunks with the highest activations and choose the action that is the most common one among those three chunks.^5^ The parser repeats this procedure until it encounters *shift*. At that moment, the parser is done with integrating word *n* and can move its attention to word *n* + 1. The activations collected during the parsing are averaged. They can be used to directly predict processing difficulties, as in section 4.2, or used to calculate reaction times, as in section 4.3.

The activation of a chunk is the sum of base-level activation and spreading activation. For base-level activation, we need to estimate how often a parsing step has been used in the past and how much time elapsed. The estimation comes from the frequency of parsing steps, collected from the PTB. The frequencies can be transformed into base-level activation according to the procedure described in Reitter et al. (2011), see also Dotlačil (2018) and Brasoveanu and Dotlačil (2020). The procedure is summarized in Appendix A.

The spreading activation is calculated based on the match between values in chunks and features in the current cognitive context at the moment when the parsing step is recalled. The features are summarized in (17).

### 4.2. Case 1: Garden-Path Sentences

We start the investigations of the predictions of the parser by considering selected garden-path phenomena, taken from previous literature (Bever, 1970; Frazier, 1978; Marcus, 1978; Gibson, 1991; Pritchett, 1992).

We model the predictions for the pairs in (18)–(21). In each pair, the (a) sentence is a classical example of a garden path. The (b) sentence carries the same or almost identical interpretation as the garden path. However, since the disambiguation takes place early in (b) sentences, no garden-path effect is observed.


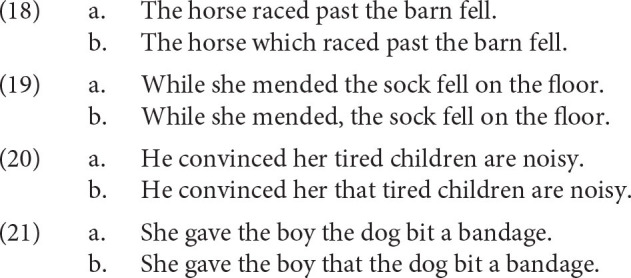


We want to see how the parser parses (18)–(21) and what activation values are predicted for the words in the sentences. We expect that the activation of the retrieved parsing steps should be lower for garden-path cases [(a) examples] compared to the (b) cases. This should happen at the target words, the words at which processing difficulties should be located in garden-path sentences. The target words are *fell* for (18), *fell* for (19), *are* for (20), and *bit* for (21). We expect the activation to decrease for garden-path sentences at the disambiguation point because the base-level activation of parsing steps should be low (garden-path sentences should not be very frequent in natural data) and because the spreading activation should be low (garden-path sentences move us to the syntactic context that cannot find a good match in the past parsing steps hence not many cues will spread activation).

**Figure 2:**
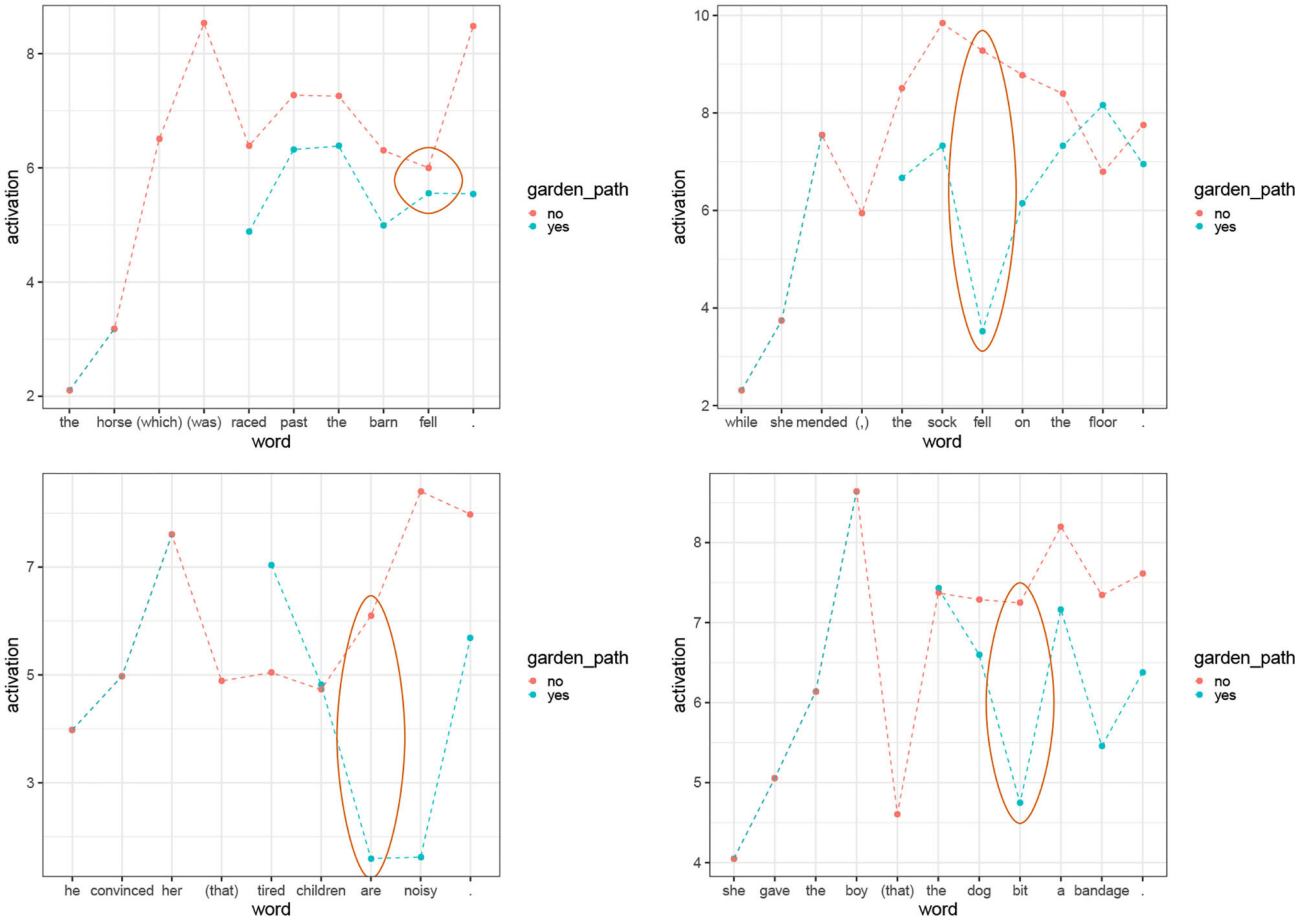
Activations per word for sentence pairs (18)–(21). The yellow bars represent the activations in the sentences that disambiguate early. The blue bars are the activations of the garden-path sentences. The ellipses highlight the activations on the words that trigger the garden-path effect.

The activations per word are graphically summarized in Figure 2. For this calculation, we assumed default values of free parameters and we set the maximum associative strength, *S*, from the Equation (8) at 20. As we can see, the (a) examples show lower activations than (b) examples at the target word. Furthermore, with one exception, the classical pair in (18), the difference not only goes in the predicted direction, but it is large at the critical word (2 points of activations or more). Note also that the contrast in activations usually spills over to the following words. Since lower activations translate into higher retrieval times we see that the model is able to predict increased reading times in garden-path sentences. Furthermore, chunks with lower activations have higher probability of retrieval failures (Anderson, 1991; Anderson and Lebiere, 1998). Consequently, the decrease in activation can explain processing difficulties in general, in particular, the failure to provide a correct parse for garden-path sentences (Pritchett, 1992).^6^

The phrase structures built by the parser are correct for all the (b) examples with the exception of (21-b) in which the parser wrongly attaches the noun phrase *a bandage* inside the relative clause. For the (a) sentences, the parser struggles at the disambiguation point and the parsing steps that it retrieves are not adequate phrase structures. It provides phrase structures that are incorrect but in which locally built phrases are combined in a plausible way. The incorrect parses for the (a) sentences were selected by the parser because they had the highest activations in the context. This means that if we restricted our attention to *correct* parses, the contrast between garden-path sentences and their (b) counterparts would be even larger at the critical words.

One pair in which the contrast between the (a) and the (b) examples goes in the right direction but is so small that the activation contrast is almost irrelevant is the case (18). The fact that the garden-path sentence almost does not differ from the baseline might be caused by the fact that we do not model discourse and semantic phenomena, while Crain and Steedman (1985) showed convincingly that this garden path is sensitive to its context. Since the model does not take context into account, it misses out on discourse effects affecting activations.

To conclude, we see that the contrasts in the activation of retrieved parsing steps can be tied to processing difficulties and predict cognitive difficulties observed in garden-path sentences.

### 4.3. Modeling Corpus Reading Data

#### 4.3.1. Introduction

We study the predictions of the parsing model for the Natural Stories Corpus (NSC, Futrell et al., 2018). The NSC is a corpus containing 10 English narrative texts with 10,245 lexical tokens in total. The texts were edited to contain various syntactic constructions, including constructions that are very rare. The corpus was read by 181 English speakers using a self-paced reading moving-window paradigm and the self-paced reading data were released along with the texts. Furthermore, all the sentences were annotated according to PTB notational conventions by the Stanford Parser (Klein and Manning, 2003) and checked and hand-corrected. The fact that the NSC has a plethora of syntactic constructions and includes manually controlled PTB-compatible syntactic parses makes the corpus particularly usable for the computational modeling of parsing.

#### 4.3.2. Reading Model

The parser as specified in sections 2 and 3 and implemented in section 4.1 will be used to model the self-paced reading of sentences in the corpus. However, to make sure that the parser does not go astray, at every word, we collect the correct parse provided by the NSC. This correct parse is used as the context for retrieval: based on this parse, the parser attempts to retrieve a parsing step from declarative memory. The declarative memory consists of parsing steps collected from the PTB, see section 4.1 for details. Then, the average activation of the retrieved chunks is recorded. After the parse for the word is finished, the correct parse is considered again for the next word. That means that the parser will have the correct syntactic structure at every word and will use the correct context for retrieval.

Importantly, in a self-paced reading task, readers do much more than just retrieving and applying parsing steps. It seems uncontroversial that a model simulating self-paced reading should, at least, attend visually to word *n*, retrieve lexical information on that word, parse, press a key (to reveal the next word) and move visual attention to the next word, word *n* + 1. We will add these parts and combine them with the parsing model to construct a more realistic model of reading. The added parts are not created *ad hoc*, they are based on the (simplified) models of visual attention and self-paced reading (Anderson and Lebiere, 1998; Brasoveanu and Dotlačil, 2020).

**Figure 3:**
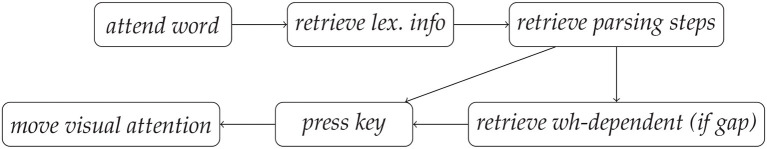
Sequential model of reading on one word. Each box represents one process. Arrows show the order in which the processes fire. There are two arrows from *retrieve parsing steps* because *retrieve wh-dependent* is only triggered when a gap is postulated by the parser.

The sequential behavior like reading is modeled in ACT-R as a case of procedural knowledge, which sequences processes, such as the ones mentioned above and calls various sub-modules (visual, declarative memory, motor module) to carry out task specifics. The processes are linked together and controlled by the procedural system. In Figure 3, we represent the processes as boxes, which the procedural system lets fire in the order as signaled by the arrows. It is assumed that these processes are repeated on every word. Firing each of these processes takes the same amount of time in the procedural system, specified in (22).


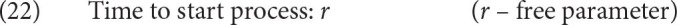


In addition to that, submodules involved in a process incur extra processing time based on their own properties.

The process *attend word* visually attends to a word. To keep the model simple, we will assume that visual attention takes a fixed amount of time, in line with basic models of ACT-R (Bothell, 2017). It is assumed that attending takes 50 ms, the default value of process firing in ACT-R. Since visual attention is modeled as a fixed amount of time, any fit of the model to the data must be driven only by retrieval processes: the retrieval of lexical information or the retrieval of syntactic information, which are the only two retrieval processes considered in this paper.

The processes *press key* and *move visual attention* interact with the motor module and the visual module, respectively. *Press key* is modeled assuming the basic model of motor actions in ACT-R, which is inspired by the EPIC cognitive architecture (Bothell, 2017). It is assumed that readers have their fingers ready on the key to be pressed. In that case, the simple model of motor actions in ACT-R, followed here, postulates that it takes 150 ms to press the key. Crucially, during this time, the procedural system is free to carry out any other actions in the sequential model. That means that moving visual attention can happen concurrently with key presses.

The processes *retrieve lex. info, retrieve parsing steps* and *retrieve wh-dependent* are the processes that depend on declarative memory. All processes take at least *r* amount of time each. Aside from that, they will also take some extra time: the amount of time needed to retrieve a chunk from declarative memory. All relevant equations to calculate retrieval time have been given in section 2. Let us repeat that the retrieval time is a function of activation of a retrieved chunk and modulated by two free parameters (23-a). Activation is calculated as the sum of base-level activation and spreading activation (23-b).


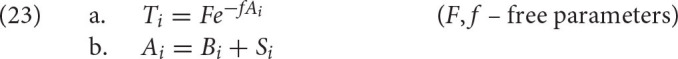


The base-level activation and spreading activation have been discussed in detail in section 2. Recall that these activations had several free parameters: decay *d*, weight *W*, maximum associative strength *S*. We set the first two parameters at their default value 0.5 and 1, respectively (see Anderson and Lebiere, 1998; Bothell, 2017). The maximum associative strength is set at 20 to ensure that associative strength is always positive (see Bothell, 2017). Furthermore, *r*, the time for the procedural system to fire a process, see (22), is set at 33 ms, as this was found in Dotlačil (accepted)^3^ to be the median value for an ACT-R model that simulates reading in a self-paced reading experiment. Finally, the time component needed to calculate base-level activation is calculated in the same way for the retrieval of lexical information (words) and the retrieval of parsing steps. It is derived from the frequencies of words and parsing steps, based on the procedure summarized in Appendix A.

This leaves us with two parameters needed to estimate retrieval times from activations: *F* and *f*. These will be estimated with a Bayesian modeling procedure.

#### 4.3.3. Bayesian Modeling

There are two parameters that we need to model to fit the reading model to the corpus data: *F* and *f*. We will estimate them using Bayesian techniques (see Dotlačil, 2018, Brasoveanu and Dotlačil, 2018, Brasoveanu and Dotlačil, 2019, Brasoveanu and Dotlačil, 2020; Rabe et al., 2021 for other examples of combining Bayesian modeling with ACT-R cognitive models; see Weaver, 2008; Dotlačil, 2018 for arguments why this is necessary).

**Figure 4:**
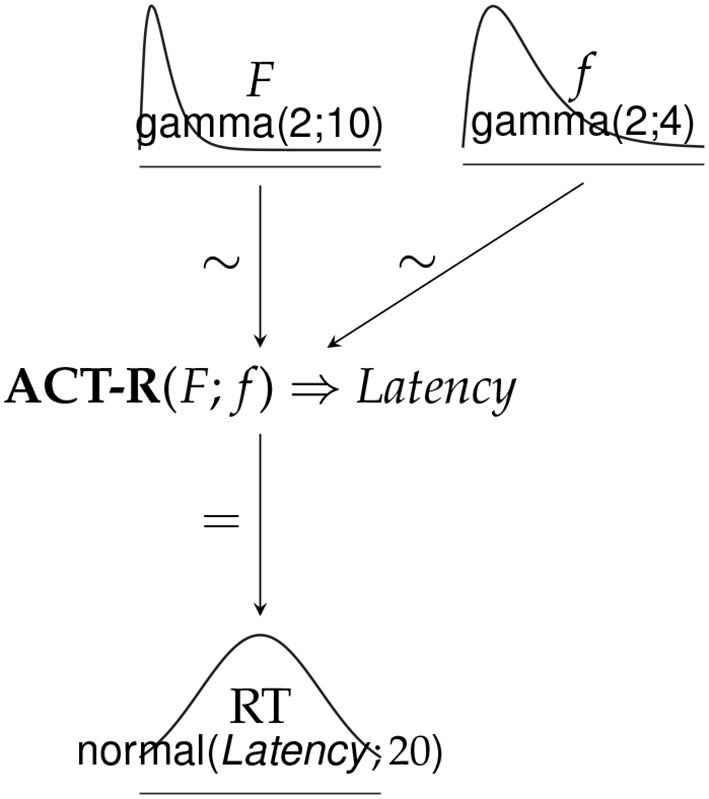
Bayesian model for parameter estimation of Natural Stories Corpus.

We assume the structure of the model as shown in Figure 4. In this graph, the top layer represents priors, the bottom part is the likelihood. **ACT-R**(F;f) is the ACT-R cognitive model of reading described in the previous section. When run and supplied with *F* and *f* values, it outputs latencies per word. The latencies of the model are then evaluated against the data assuming the likelihood is a normal distribution (measured in milliseconds) with standard deviation 20 ms (the bottom part of the graph). The actual data that we try to model are mean reading times (mRT) per word in the self-paced reading corpus. We select the first two (out of 10) stories for the estimation of the parameters. In each story, there is an observable effect of speed-up as readers progress beyond the first few sentences. Since our model does not represent that, we decided to remove the first 10 sentences from each story. Furthermore, we model mRTs only starting at the second word and ending at the second to last word in each sentence since the first and last words tend to be outliers due to starting and wrap-up effects. Besides, the starting words are also outliers in our model (see also text footnote 6).

The following prior structure for the parameters is assumed:

- *F* ~ *Gamma*(α = 2, β = 10)
- *f* ~ *Gamma*(α = 2, β = 4)

Given these priors, the values in the range 0–1 are most likely but extremely low values are penalized. The priors for the parameters have the mean values of 0.2 and 0.5, respectively. These priors take into account previous findings that when *F* and *f* are estimated on language studies, including reading data, they are below 1 but usually not exceedingly small and *F* tends to be smaller than *f* (Brasoveanu and Dotlačil, 2018, 2020).

The estimation of parameters was done using pymc3 and MCMC-sampling with 1,200 draws, 2 chains and 400 burn-in draws. The sampling chains converged as witnessed by the Rhat value (Rhat for *F* was 1.036; Rhat for *f* was 1.028).

#### 4.3.4. Results

The mean, median and standard deviation values for the latency factor (*F*) and latency exponent (*f*) of the posterior distributions can be seen in Table 1.

**Table 1:** Estimated parameter values.

	**Mean**	**Median**	**Std**
*F*	0.0139	0.0139	0.001
*f*	0.661	0.655	0.068

The mean and median values for *F* match the estimate in previous Bayesian + ACT-R reading models (Brasoveanu and Dotlačil, 2018, 2020). However, the estimate of *f* is greater than in previous reading studies. It is possible that this is because the previous reading studies did not take the retrieval of parsing steps into account, focusing only on lexical retrieval and that the previous studies mainly looked at experimental data, while this study models corpus data.

To further investigate the model, we check samples from its posterior distribution of predicted RTs (i.e., RTs that the reading model predicts using the posterior distribution of the fitted parameters). We expect that these should correlate with observed meanRTs. This is because the model simulates two steps in processing, namely, lexical retrieval and parsing. Lexical retrieval is affected by the activation of words, which depends on frequency and causes less frequent words take more time to retrieve than more frequent words (see Appendix A for the estimation of base-level activation based on frequency). Syntactic retrieval is affected by the activation of parsing steps, which is the sum of base-level activation and spreading activation. The base-level activation is related to frequency just like word activation and makes less frequent parsing steps take more time to retrieve (see Appendix A). Furthermore, if a reader is in a rare syntactic context (i.e., an uncommon syntactic construction), they are less likely to find parsing steps in the past that would provide a good match. This results in a decreased spreading activation, which again affects reading times. Finally, the parser models wh-dependency and retrieving wh-words will increase reading times when the wh-words are far away from the gap site, due to the decrease in their activation.

We now inspect the predictions of the model. First, we run a simple linear model with predicted RTs per word (i.e., RTs that the reading model predicts using the posterior distribution of the fitted parameters) as the independent variable and observed mean RTs as the dependent variable. We see in the summary of the linear model given in Table 2 that the Maximum Likelihood Estimate (MLE) of predicted RT is very close to 1, i.e., in the best linear fit between the predicted and observed RT, the increase of 1 ms in predicted RTs corresponds to the increase of 1 ms in observed RTs. Table 3 shows the fit of the intercept + predicted RT linear model. As we see, predicted RTs are a highly significant predictor for observed mean RTs.

**Table 2:** The linear model with Predictive RT as the only independent variable.

	**Estimate**	**SE**	***t*-Value**	***p*-Value**
Predictive RT	0.993	0.0024	415.5	*p* < 0.0001

**Table 3:** The linear model with Intercept and Predictive RT.

	**Estimate**	**SE**	***t*-Value**	***p*-Value**
Intercept	248.4	12.7	19.57	*p* < 0.0001
Predicted RT	0.220	0.040	5.55	*p* < 0.0001

The finding in Table 3 shows that our reading model can capture some aspects of self-paced reading data. However, we want to see that this modeling capability goes beyond what surface features of a text, i.e., position, word length or string frequencies, known to influence reading times, can account for. For this reason, we consider a more complex model, summarized in Table 4. The confounds we consider are the following: (i) Story (story 1 or story 2, the former being the reference level), (ii) Zone (the word position in its story, z-transformed), (iii) Position (the word position in its sentence, z-transformed), (iv) the interaction of Story × Zone, (v) the interaction of Zone × Position, (vi) Log(Freq) (log-unigram frequency), (vii) Nchar (the length of the word in number of characters, z-transformed), (viii) the interaction of Nchar × Log(Freq), (ix) Log(Bigram) (log bigram probability), (x) Log(Trigram) (log trigram probability). Frequencies and bigram and trigram probabilities are provided in the NSC. Most of the confounds that we input are considered when evaluating computational psycholinguistic models on corpus data (Demberg and Keller, 2008; Boston et al., 2011; Hale, 2014, among others). We see that even after adding the confounds, predicted RTs remain a significant predictor and the effect goes in the expected (positive) direction (*t* = 3.66, *p* = 0.0003). Thus, our parsing model captures aspects of reading data that are not captured by surface-like factors, e.g., string frequencies, position, number of characters and the interaction of those.^7^

**Table 4:** A full linear model for RTs in the NSC.

	**Estimate**	**SE**	***t*-Value**	***p*-Value**
Intercept	258.5	17.2	15	*p* < 0.0001
Story	7.3	1.3	5.5	*p* < 0.0001
Zone	−3.9	0.87	−4.5	*p* < 0.0001
Position	−2	0.7	−3	0.003
Story:Zone	−3.3	1.34	−2.5	0.01
Zone:Position	1.65	0.73	2.25	0.02
Nchar	16.3	3.79	4.3	*p* < 0.0001
Log(Freq)	0.21	0.52	0.4	0.7
Nchar:log(Freq)	−0.68	0.22	−3.1	0.002
Log(Bigram)	0.25	0.63	0.4	0.7
Log(Trigram)	−0.88	0.48	−1.82	0.07
Predicted RT	0.15	0.04	3.66	0.0003

**Figure 5:**
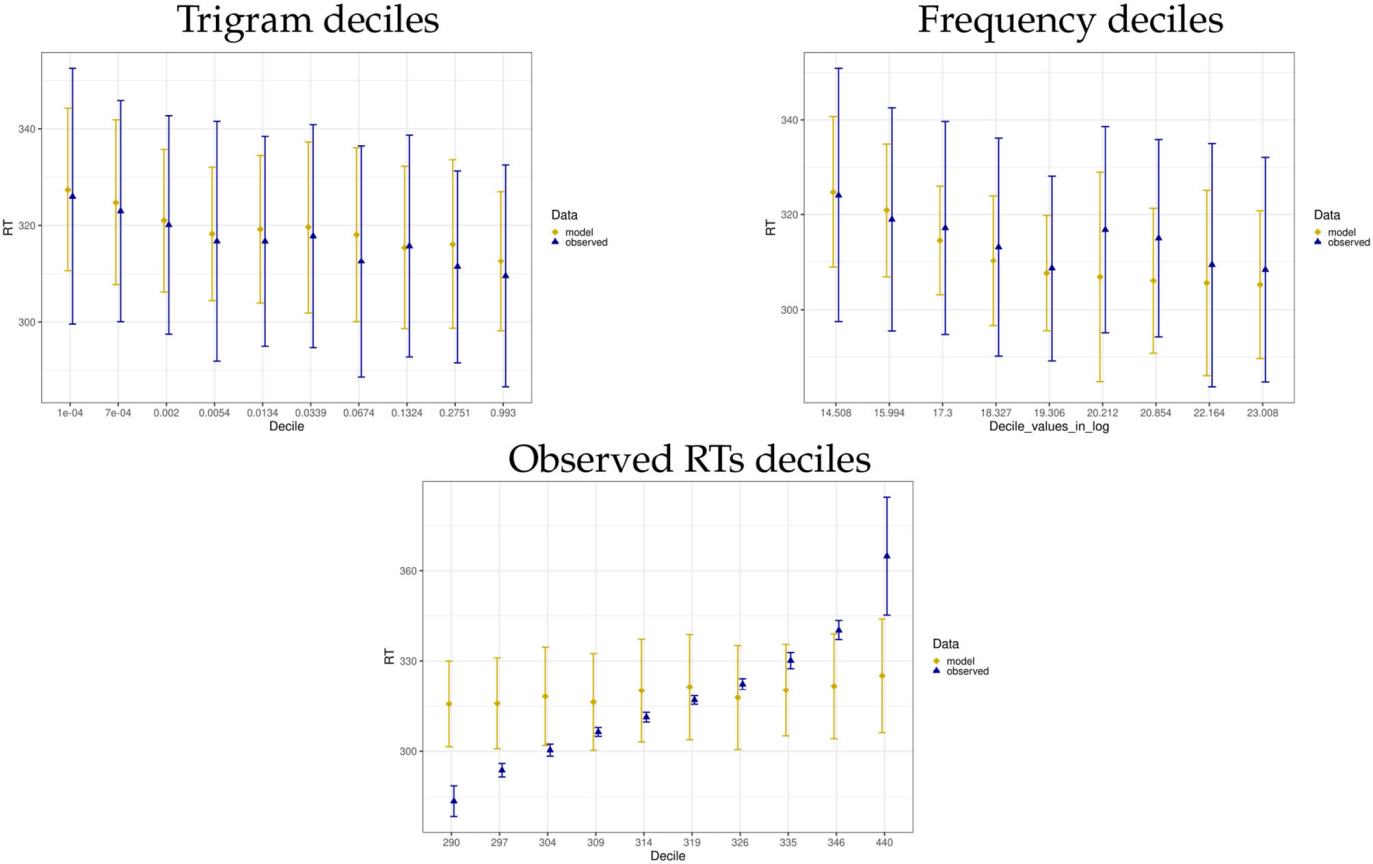
Mean and standard deviation summaries of model and data split per trigram, frequency and observed mean RT deciles. The x-axis label shows the upper cut-off point per decile (given in log in case of Frequency). In case of Frequency, only 9 deciles are present. This is because a single word (*the*) spans the top two deciles.

To further inspect the predictions of our Bayesian + ACT-R model and the actual data, we split the predicted and observed data sets into deciles based on trigrams, word frequencies and the actual observed mean RTs. The graphical summaries per decile are given in Figure 5. For trigram probabilities and unigram frequencies, we see that the data predicted by the model follows the trend of the actual data and the mean predicted RT is generally close to the observed mean RT in each decile (with the slight divergence in the 6th and 7th decile of Frequency, for which the model assumes mean RTs faster by 10 and 9 ms). In case of the last graph, in which data are split by observed mean RT deciles, the model copies the linear trend of the data, i.e., predicted mean RTs increase per decile. This trend is also confirmed by a highly significant Pearson correlation between predicted mean RT and observed mean RT split by decile (*r* = 0.88, *p* < 0.001). However, compared to the actual data, the model has much less extreme values on both ends of the decile spectrum and as the result. While it captures the linear trend in the data, it overestimates RTs in low deciles and underestimates RTs in high deciles.

Finally, we compare the predictions of our model to another ACT-R model of reading, presented in Boston et al. (2011). The model of Boston et al. (2011) models the retrieval of dependencies using the assumptions of the ACT-R rational memory. In contrast to our work, Boston et al. (2011) do not model structure building, i.e., the knowledge of parsing steps, using the ACT-R memory.^8^ For this reason, we would expect that the time predictions of our model remain a significant predictor when the predictions of Boston et al. (2011) are included in a linear model of the NSC reading data. To check this, we constructed time predictions of the ACT-R reading model of Boston et al. (2011) for the NSC sub-corpus that we used for testing (the first two stories).^9^ We tested the ACT-R retrieval model of Boston et al. (2011) with various levels of beam-width *k* (*k* = 1, 3, 9, 20, 50, 100), where *k* specifies the number of syntactic parses built in parallel. It turned out that model predictions with low numbers of *k* (*k* ≤ 20) did not show a significant effect on our NSC reading data. For *k* = 50 and *k* = 100, the model showed a very wide range of predicted reading times (from 50 to 5,000 ms). When we removed predictions beyond 2,000 ms, the model predictions were significant (β = 0.005, *t* = 3.1). Crucially, the predictions of our model, Predicted RT, were also significant (β = 0.2, *t* = 4.1). This supports the position that our model captures the properties of reading missing in an ACT-R model that only simulates the retrieval of dependencies using the ACT-R theory of memory.

### 4.4. Summary of the Results

We provided empirical evidence for the parsing model that is built on the assumptions of the rational theory of memory proposed in Anderson (1991) and embedded in ACT-R. Two types of evidence were collected. First, processing difficulties of garden-path phenomena correspond to activation drop of retrieved parsing steps. Second, the parsing model, combined with some basic assumptions about reading, has been used to model self-paced reading data from the Natural Stories Corpus. After fitting two parameters, the resulting model showed a highly significant correlation with observed reading times. The model was able to capture aspects of the reading data that were not captured by other, low-level factors like string frequencies, position or word length. We leave it open which particular aspects of the rational memory might play a dominant role in model fitting, in particular, which of base-level activation and spreading activation was crucial in our finding.

## 5. Comparison to Related Works

### 5.1. Parsers in Computational Psycholinguistics

It is possible to split the computational psycholinguistic approaches to parsing into two types, experience-based theories and memory-based theories. In experience-based theories, it is studied how past experience with syntactic structures affect parsing, most often because of expectations readers form during sentence processing. A popular framework belonging to experience-based approaches is Surprisal Theory (Hale, 2001; Boston et al., 2008, 2011; Levy, 2008, 2011; Smith and Levy, 2013, among others). In memory-based theories, it is studied how the bottleneck of memory affects storage and retrieval during processing. Dependency Locality Theory is an example of a memory-based explanation of processing difficulties (Gibson, 1998), and so are theories studying the effect of integration and recall of information from parsing stacks (Van Schijndel and Schuler, 2013; Shain et al., 2016; Rasmussen and Schuler, 2018). Another memory-based theory is the activation-based approach to dependency resolution, often implemented in ACT-R (see Lewis and Vasishth, 2005; Lewis et al., 2006).

The two types of approaches offer different advantages. While experience-based theories can account for processing difficulties tied to construction frequency and local ambiguities (garden-path phenomena), memory-based approaches are used to capture locality effects. However, the integration of the two accounts into one framework is arguably still an open issue. In most accounts, two research lines are simply put together as two different and separated parts of a model (Demberg and Keller, 2008; Boston et al., 2011; Levy et al., 2013; Van Schijndel and Schuler, 2013).

In contrast to the just cited approaches, the current account builds a single analysis of experience-driven and memory-driven processing difficulties. It is assumed that both difficulties are driven by memory limitations in retrieval, as predicted by rational memory systems. The only difference is what is being retrieved: memory-driven processing difficulties arise when the memory system tries to recall a recently constructed phrase/element to satisfy dependency and encounters problems; experience-driven difficulties arise when *the same* memory system tries to recall a parsing step and encounters problems. The first type of difficulties has been well-investigated in computational psycholinguistics in general and in the sub-field of modeling using cognitive architectures like ACT-R in particular (see Lewis and Vasishth, 2005; Lewis et al., 2006; Dubey et al., 2008; Reitter et al., 2011; Engelmann et al., 2013; Engelmann, 2016; Vogelzang et al., 2017; Brasoveanu and Dotlačil, 2020). Crucially, the second type of difficulties has been investigated much less from this perspective. This paper can be seen as an attempt to enhance our understanding on this topic. In this respect, this paper advances current ACT-R analyses of reading, notably Lewis and Vasishth (2005), which do not generalize parsing, relying instead only on hand-coded rules for selected syntactic constructions. An account that offered one framework for both types of processing difficulties has been developed in Futrell and Levy (2017), which provides a computational-level analysis (in contrast to the algorithmic-level analysis developed here) and comes to the problem from the opposite direction. Futrell and Levy (2017) provides a single analysis to processing difficulties by expanding Surprisal Theory with an extra component (noisy-context) to capture memory-driven difficulties.

In works within cognitive architectures, a close affinity can be found between this account and the models of Reitter et al. (2011) and Hale (2014).

Unlike Reitter et al. (2011), the current account does not model production, but focuses on comprehension, and it does not study priming of syntactic rules. Furthermore, Reitter et al. (2011) developed a model to generate qualitative effects in priming, while this paper shows that, through the application of ACT-R models in a Bayesian framework, it is possible to model quantitative data patterns. In fact, the presented approach makes it possible to develop a model in which the reading profile of experience-driven processing difficulties quantitatively constrains the reading profile of memory-driven processing difficulties, since both phenomena are modeled in the same way and modulated by the same free parameters. This has also been assumed in this paper (e.g., the parser for the Natural Stories Corpus assumes the same model for retrieval of wh-dependency, lexical retrieval and the retrieval of parsing steps). However, a close investigation of the interaction of different cases of retrieval in the same model goes beyond the scope of this paper. See Dotlačil (accepted)^3^ for more work in this direction.

Finally, Hale (2014), Chapters 7 and 8, derives experience-driven processing difficulties as a case of (failed, less likely) production compilation/cohesion. This position is not incompatible with the current account, in fact, it complements it. While this work studies the role of declarative memory on parsing, Hale (2014) focuses on the role of procedural memory on parsing. The latter position has arguably been investigated in much more detail in psycholinguistics and in ACT-R than the former position since the seminal works of Lewis (1993) and Lewis and Vasishth (2005). In this respect, the current proposal can be seen as breaking with this tradition. However, both types of memory are crucial for ACT-R as well as other cognitive architectures (see Anderson, 2007) and their interaction is needed to account for complex learning patterns (Lebiere, 1999; Taatgen and Anderson, 2002). It is likely that a highly non-trivial task, such as syntactic structure-building will benefit from investigations that do not limit its investigation to the procedural memory system.

### 5.2. Transition-Based Parsing in Computational (Psycho)linguistics

Transition-based parsers were a popular choice of parsers in computational linguistics, especially for dependency grammars (see Nivre et al., 2007; Zhang and Clark, 2008; Kübler et al., 2009). One advantage of transition-based parsers over graph-based parsing and grammar-based parsing is that they are fast, incremental and they allows for rich feature representations (Nivre, 2004; McDonald and Nivre, 2011). Transition-based parsers have also been applied to phrase-structure parsing (Kalt, 2004; Sagae and Lavie, 2005). The recent neural transition-based parsers for phrase-structure building have the F1 value around 95% on the PTB section 23 (Liu and Zhang, 2017; Kitaev and Klein, 2018). Transition-based parsers have also been used in computational psycholinguistics to model EEG data (Recurrent neural network grammars; Dyer et al., 2016; Hale et al., 2018) and reading data (Boston et al., 2008; Rasmussen and Schuler, 2018).^10^

While the high accuracy of the state-of-the-art transition-based parsing is encouraging, as it suggests that this line of parsing can eventually be used to create a very accurate parser, we should note that our parser is nowhere near this accuracy performance. When tested on the section 23 of the Penn Treebank, the parser shows Label Precision as 70.2, Label Recall as 72.4, F1 as 71.3. When we restrict attention to sentences of 40 words or less, as is common, Label Precision is 73.7, Label Recall is 75.9, and F1 is 74.8.^11^

There are arguably several reasons for the low performance. First, it has been found that one of the disadvantages of transition-based parsers when compared to another class of data-driven parsers, graph-based parsers, is that they get worse with increase in sentence length and increase in dependence, i.e., error propagation (McDonald and Nivre, 2011). Traditional transition-based parsers, including the parser in this paper, explore just one path. They have to greedily select what path they will follow and stick to it until the end of the sentence. Thus, early mistakes will propagate the error throughout the whole sentence. Better transition-based parsers mitigate this type of mistake through beam search or methods to recover from errors. While the adaptation of these methods could be investigated for psycholinguistics, we are not primarily interested in the best accuracy of the parser on the complex Penn Treebank sentences, but in parsing that is human-like. It is known that a human processor also shows error propagation in parsing, as witnessed by the fact that readers struggle to recover from garden path sentences the longer the wrong interpretation can be held (e.g., Frazier and Rayner, 1982). Thus, it is not a priori clear that error propagation should be avoided.

Another reason why we see a low accuracy is that the parser assumes a very straightforward relation between memory instances and a parsing step. A parsing step is simply stored in declarative memory.^12^ This is in contrast to complex training methods commonly assumed in current neural parsers. Relatedly, current computational parsers assume a much richer feature system. They are enriched by vector space models representing lexical information and syntactic information is usually encapsulated in 200 or more features, while our parser has 19 features.

In any case, it might be worth pointing out that even though the accuracy of the parser is not very high, it suffices for the research presented in this paper. The chosen examples in section 4.2 are correctly constructed by the parser when they do not lead to garden path and the parser in section 4.3 was at the end of every step (word) corrected to match the gold standard provided in the corpus, ensuring that the constructed parse is correct.

The decision to have a simple feature model is driven by the fact that we want to first establish that this model of parsing can be useful in predicting reading times. For that, it is preferable to keep the model as comprehensible and simple as possible, otherwise, it would not be clear whether the results reported in section 4 are due to the parsing model or some confound we are not interested in (e.g., meaning similarity present in word vector spaces). For the same reason, we currently made use of the bottom-up parsing algorithm, even though there is a good argument to be made that the bottom-up parsing algorithm is not cognitively adequate. There are well-known issues with bottom-up parsing for psycholinguistics: it accumulates elements on the stack in right-branching structures, suffers from disconnectedness and has problems when tied to incremental interpretation (see Resnik, 1992; Crocker, 1999). We assumed the bottom-up parsing algorithm since it is arguably the most common parsing algorithm for transition-based phrase structure parsers and thus, it serves as a very good starting point. We leave it for the future to see whether other parsing algorithms, notably, left-corner parsers, can improve on the current modeling results.

## 6. Conclusion

This paper presented and tested a psycholinguistic parser that has been developed using insights from the rational theory of memory. It has been shown that the rational theory of memory can be combined with transition-based parsing to produce a data-driven parser that can be embedded in the ACT-R cognitive architecture. The parser has been tested on garden-path sentences and it has been shown that the parser to a large extent predicts processing difficulties at correct disambiguation points. The parser has also been evaluated on on-line behavioral data from a self-paced reading corpus and it has been shown that the parser can be fit to data and model quantitative patterns in reading times.

## Data Availability Statement

The raw data supporting the conclusions of this article are available at https://github.com/jakdot/parsing-model-and-a-rational-theory-of-memory.

## Author Contributions

JD contributed to the development of the theory, coding, and modeling. PH contributed to coding and modeling.

## Conflict of Interest

The authors declare that the research was conducted in the absence of any commercial or financial relationships that could be construed as a potential conflict of interest.

## Footnotes

^1^The procedural memory system stores the knowledge exhibited in automatized, sequential behavior. This type of memory plays less important role in our models of parsing. We will briefly come back to it in section 4.

^2^ACT-R activation also standardly includes noise parameter. In (5), we ignore the noise parameter ϵ, so that activation is deterministic.

^3^Dotlačil, J. (accepted). Parsing as a cue-based retrieval model. *Cogn. Sci*.

^4^The code for the model is available here: https://github.com/jakdot/parsing-model-and-a-rational-theory-of-memory.

^5^Using three chunks, rather than a single chunk, to inform about the action, makes the parser less error-prone and sensitive to outliers. Adding more than three chunks does not improve the accuracy of the parser. We briefly discuss the accuracy of the parser in section 5.

^6^The activations are also very low at the beginning of each sentence, irrespective of whether we deal with a garden-path sentence or not. This is an artifact of the selected model. Most cues for spreading activation come from the tree structures already built. Of course, nothing or almost nothing has been built at the beginning of a sentence, hence there are few cues at the start and consequently, spreading activation is low. It is possible to avoid this property of the model, for example, by not counting just matches in built trees, but also matches by the position in a sentence as cues that can boost activations.

^7^It might seem surprising that the effect of log-frequency is not significant in Table 4. This is because predicted RTs correlate with frequency and because we also include the Nchar × Log(Freq) interaction. In a simpler model lacking the interaction, Log(Freq) is significant and goes in the expected direction.

^8^See also section 5 for comparisons of our model to related works.

^9^We used the code available at https://conf.ling.cornell.edu/Marisa/. To generate predictions, we made use of the default English training corpus, Brown. We would like to thank an anonymous reviewer, Marisa Boston and John Hale for discussion and help.

^10^While the mentioned works in computational psycholinguistics make use of transition-based parsing, they are not closely related to this work. The cited approaches, unlike the current account, do not construct the parsers inside a cognitive architecture and their goal is different than developing a single account for experience-based and memory-based processing difficulties based on the rational theory of memory.

^11^Label Precision is calculated as the number of correctly constructed constituents divided by the number of all constituents proposed by the parser. Label Recall is calculated as the number of correctly constructed constituents divided by the number of all constituents present in the gold standard. F1 is the harmonic mean of the two accuracy measures. For the calculation, only non-terminal constituents are used for accuracy (i.e., trivial constituents like 〈*a, DT*〉 are ignored so that the accuracy measures are not artificially inflated).

^12^The parser could be subsumed under a case of memory-based parsing, see Daelemans et al. (2004). However, unlike the past cases of memory-based parsing, which were inspired by memory structures to deliver the best accuracy on data-driven parsing, the current approach is inspired by memory structures to connect parsing to on-line behavioral measures. Such a link is not considered in the approach of Daelemans et al. (2004).

## Appendix A: Calculate Base-Level Activation From Word/Rule Frequencies

We want to calculate *B*_*i*_ from frequency. *d* is a free parameter and can be ignored in this discussion.

Consider a 15-year old speaker. How can we estimate how often a word/parsing step *x* was used in language interactions that the speaker participated in?

First, let's notice that we know the relative frequency of *x*. We collect that from the British National Corpus (for words) and from the Penn Treebank corpus (for parsing steps).

We know the lifetime of the speaker (15 years), so if we know the total number of words an average 15-year old speaker has been exposed to, we can easily calculate how many times *x* was used on average based on the frequency of *x*. A good approximation of the number of words a speaker is exposed to per year can be found in Hart and Risley (1995). Based on recordings of 42 families, Hart and Risley estimate that children comprehend between 10 million to 35 million words a year, depending to a large extent on the social class of the family, and this amount increases linearly with age. According to the study, a 15-year old has been exposed to anywhere between 50 and 175 million words total. For simplicity, the model will work with the mean of 112.5 million words as the total amount of words a 15-year old speaker has been exposed to. This is a conservative estimate as it ignores production and the linguistic exposure associated with mass media. Furthermore, we assume that each word is accompanied by one parsing step, so there are as many parsing steps as words (again, this is a simplification that should not harm modeling).

We now know how we get from frequency to the number of usages of *x*. Simplifying again, we assume that the usages, *t*_*k*_ above, are evenly spread during the life span.

The procedure described here was successfully used in translating frequencies to activations and ultimately reaction times in sentence production (Reitter et al., 2011), eye tracking reading times (Dotlačil, 2018) and reaction times in lexical decision tasks (Brasoveanu and Dotlačil, 2020).
